# Cytokines mapping for tissue-specific expression, eQTLs and GWAS traits

**DOI:** 10.1038/s41598-020-71018-6

**Published:** 2020-09-07

**Authors:** Lyubov E. Salnikova, Maryam B. Khadzhieva, Dmitry S. Kolobkov, Alesya S. Gracheva, Artem N. Kuzovlev, Serikbay K. Abilev

**Affiliations:** 1 Laboratory of Ecological Genetics, N.I. Vavilov Institute of General Genetics, Russian Academy of Sciences, 3 Gubkin Street, Moscow, Russia 117971; 2 Laboratory of Clinical Pathophysiology of Critical Conditions, Federal Research and Clinical Center of Intensive Care Medicine and Rehabilitology, Petrovka str, 25, b.2, Moscow, Russia 107031; 3 Department of Computer Science and Applied Mathematics, Weizmann Institute of Science, 234 Herzl St., PO Box 26, 7610001 Rehovot, Israel; 4 Department of Molecular Cell Biology, Weizmann Institute of Science, 234 Herzl St., PO Box 26, 7610001 Rehovot, Israel

**Keywords:** Genetics, Gene expression, Genetic association study, Genotype, Immunogenetics

## Abstract

Dysregulation in cytokine production has been linked to the pathogenesis of various immune-mediated traits, in which genetic variability contributes to the etiopathogenesis. GWA studies have identified many genetic variants in or near cytokine genes, nonetheless, the translation of these findings into knowledge of functional determinants of complex traits remains a fundamental challenge. In this study we aimed at collection, analysis and interpretation of data on cytokines focused on their tissue-specific expression, eQTLs and GWAS traits. Using GO annotations, we generated a list of 314 cytokines and analyzed them with the GTEx resource. Cytokines were highly tissue-specific, 82.3% of cytokines had Tau expression metrics ≥ 0.8. In total, 3077 associations for 1760 unique SNPs in or near 244 cytokines were mapped in the NHGRI-EBI GWAS Catalog. According to the Experimental Factor Ontology resource, the largest numbers of disease associations were related to ‘Inflammatory disease’, ‘Immune system disease’ and ‘Asthma’. The GTEx-based analysis revealed that among GWAS SNPs, 1142 SNPs had eQTL effects and influenced expression levels of 999 eGenes, among them 178 cytokines. Several types of enrichment analysis showed that it was cytokines expression variability that fundamentally contributed to the molecular origins of considered immune-mediated conditions.

## Introduction

Cytokines are regulatory proteins and glycoproteins that are synthesized and secreted by immune system cells and other cell types. They regulate innate and acquired immunity, embryogenesis, hematopoiesis, inflammation and regeneration processes, and proliferation. These functions are realized through cell signaling and intercellular communication. Cytokines may act via autocrine manner, if they stimulate their own secretion; paracrine, if they have an effect on adjacent cells; or endocrine, if they diffuse to distant regions of the body. Cytokines function through binding to specific receptors, which send signals to recipient cells. Cytokines may also affect the expression of receptors, which in turn may influence the responsiveness of both secreting cells and target cells. Generally, cytokines are pleiotropic, i.e. have many overlapping functions, and redundant, i.e., each function is mediated by more than one cytokine. The complexity of cytokine interactions is defined as the “cytokine network”^1,2^.

The basal and stimulus-induced expression of cytokines is under tight genetic control and strongly varies between individuals^3^. Dysregulation in cytokine production has been linked to the pathogenesis of various immune deficiencies, acute and chronic infections and many chronic conditions, in particular autoimmune diseases, allergic diseases, and malignancies, all disorders in which genetic variability contributes to the etiopathogenesis. The genome-wide association studies (GWAS) have identified many genetic variants in or near cytokine genes, nonetheless, the translation of these findings into knowledge of functional determinants of complex immune-related traits remains a fundamental challenge^4^. Linking nucleotide sequences with the disease genes through expression quantitative trait loci (eQTL) analysis may help to identify the tissue-specific effects and mechanisms associated with human disease phenotypes^5^.

In this study, we characterized tissue-specificity of cytokines, analyzed eQTLs influencing expression of individual cytokines and their clusters and summarized GWAS data for cytokine associations. Since a GWAS signal may be due to a synthetic association provided by a rare high-effect variant in linkage disequilibrium (LD) with a common SNP^6^, we compared functional annotations and functional scores for index SNPs and LD SNPs. We applied two natural selection tests to identify GWAS cytokine SNPs under positive selection. Finally, we selected GWAS SNPs with eQTL activity and characterized their target gene spectrum and possible implication in diseases via their influence on cytokine expression in disease-relevant tissues.

## Results

### The overall study design

**Figure 1:**
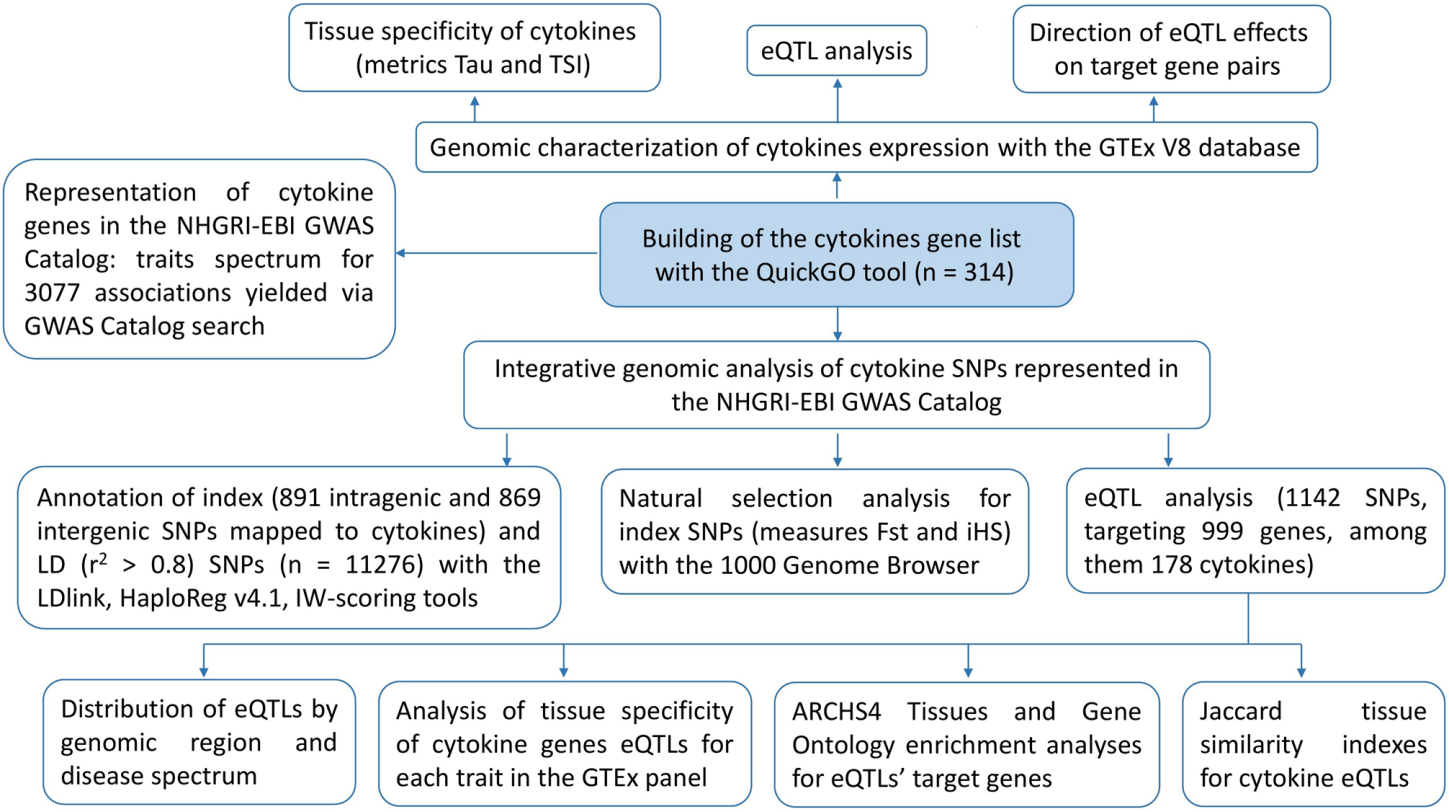
Flowchart of the study design.

The flowchart of study design is shown in Fig. 1. After building the list of genes encoding proteins referred to as cytokines, we performed genomic characterization of cytokines expression, which included the analysis of expression tissue specificity, genome-wide detection of cytokine eQTLs, and examination of the direction of eQTL effects on target gene pairs. Next, we described the phenotypic spectrum of cytokine gene associations in the NHGRI-EBI GWAS Catalog and conducted an integrative genomic investigation of cytokine SNPs represented in the NHGRI-EBI GWAS Catalog. This investigation consisted of the following steps: compiling the lists of GWAS trait-associated SNPs (index SNPs) and their LD SNPs, functional annotation of index and LD SNPs, natural selection analysis, and eQTL analysis of GWAS Catalog SNPs in cytokine genes. To identify functional effects of GWAS-identified variants by the means of eQTL analysis, we analysed the distribution of eQTLs by genomic region and disease spectrum, performed the analysis of tissue specificity of cytokine genes eQTLs for each trait in the GTEx panel, conducted ARCHS4 Tissues and Gene Ontology enrichment analyses for eQTLs’ target genes, and calculated Jaccard tissue similarity indexes for cytokine eQTLs. This last item was aimed at establishing the role of cytokine eQTLs in cytokine expression tissue specificity.

### The list of genes encoding proteins with cytokine and cytokine receptor activity

The list of 314 genes encoding proteins with cytokine/chemokine activity and cytokine/chemokine receptor activity (combined under the name ‘cytokines’) was constructed with the QuickGo tool^7^ (Table 1, Supplementary Table S1). The terms cytokine activity (GO:0005125), chemokine activity (GO:0008009), cytokine receptor activity (GO:0004896) and chemokine receptor activity (GO:0004950) yielded, correspondingly, 219, 50, 70 and 25 genes. Two types of activities for corresponding proteins were identified for 51 genes. We then classified cytokines on the basis of having growth factor activity (GO:0008083). It was attributed to 68 genes, of which 67 genes encoded proteins with cytokine activity.

**Table 1:** Genes regulating proteins with cytokine activity (GO:0005125), chemokine activity (GO:0008009), cytokine receptor activity (GO:0004896) and chemokine receptor activity (GO:0004950).

Cytokine activity (n = 170)
*ADIPOQ, AIMP1,* *AREG, *BMP1, *BMP10, *BMP15, *BMP2, *BMP3, *BMP4, *BMP5, *BMP6, *BMP7, *BMP8A, *BMP8B, C1QTNF4, CD40LG, CD70, CER1, *CLCF1, CMTM1, CMTM2, CMTM3, CMTM4, CMTM5, CMTM6, CMTM7, CMTM8, *CNTF, *CSF1, *CSF2, *CSF3, CTF1, EDN1, *EPO, FAM3B, FAM3C, FAM3D, FASLG, *FGF2, FLT3LG, *GDF1, *GDF10, *GDF11, *GDF15, *GDF2, *GDF3, *GDF5, *GDF6, *GDF7, *GDF9, *GPI, GREM1, GREM2, *GRN, HMGB1, IFNA1, IFNA10, IFNA13, IFNA14, IFNA16, IFNA17, IFNA2, IFNA21, IFNA4, IFNA5, IFNA6, IFNA7, IFNA8, IFNB1, IFNE, IFNG, IFNK, IFNL1, IFNL2, IFNL3, IFNL4, IFNW1, *IL10, *IL11, *IL12A, IL13, IL15, IL16, IL17A, IL17B, IL17C, IL17D, IL17F, IL18, IL19, IL1A, IL1B, IL1F10, IL1RN, *IL2, IL20, IL21, IL22, IL23A, IL24, IL25, IL26, IL27, *IL3, IL31, IL32, IL33, *IL34, IL36A, IL36B, IL36G, IL36RN, IL37, *IL4, *IL5, *IL6, *IL7, *IL9, *INHA, *INHBA, *INHBB, *INHBC, *INHBE, *KITLG, *LEFTY1, *LEFTY2, *LIF, LTA, LTB, MIF, *MSTN, *MYDGF, NAMPT, *NDP, *NODAL, *NRG1, *OSM, SCG2, SCGB3A1, SECTM1, SLURP1, SPP1, *TGFB1, *TGFB2, *TGFB3, THNSL2, *THPO, *TIMP1, TNF, TNFRSF11B, TNFSF10, TNFSF11, TNFSF12, TNFSF12-TNFSF13, TNFSF13, TNFSF13B, TNFSF14, TNFSF15, TNFSF18, TNFSF4, TNFSF8, TNFSF9, TSLP, TXLNA, *VEGFA, VSTM1, WNT1, WNT2, WNT5A, WNT7A
Cytokine and chemokine activity (n = 46)
*C10orf99, CCL1, CCL11, CCL13, CCL14, CCL15, CCL15-CCL14, CCL16, CCL17, CCL18, CCL19, CCL2, CCL20, CCL21, CCL22, CCL23, CCL24, CCL25, CCL26, CCL27, CCL28, CCL3, CCL3L1, CCL4, CCL4L1, CCL4L2, CCL5, CCL7, CCL8, CKLF, CX3CL1,* *CXCL1, CXCL10, CXCL11, *CXCL12, CXCL13, CXCL14, CXCL16, CXCL2, CXCL6, CXCL8, CXCL9, PF4, *PPBP, XCL1, XCL2
Chemokine activity (n = 4)
*C5, CXCL3, CXCL5, PF4V1*
Cytokine receptor activity (n = 65)
*CD4, CD44, CD74, CNTFR, CRLF2, CSF2RA, CSF2RB, CSF3R, EPOR, F3, FLT3, GFRA1, GFRA2, GFRA4, GFRAL, GHR, IFNAR1, IFNAR2, IFNGR1, IFNGR2, IFNLR1, IL10RA, IL10RB, IL11RA, IL12RB1, IL12RB2, IL13RA1, IL13RA2, IL15RA, IL17RA, IL17RB, IL17RC, IL17RD, IL17RE, IL17REL, IL18R1, IL1R1, IL1R2, IL1RAP, IL1RAPL2, IL1RL1, IL1RL2, IL20RA, IL20RB, IL21R, IL22RA1, IL22RA2, IL23R, IL27RA, IL2RA, IL2RB, IL2RG, IL31RA, IL3RA, IL4R, IL5RA,* *IL6R, IL6ST, IL7R, IL9R, LEPR, LIFR, MPL, OSMR, PRLR
Cytokine and cytokine receptor activity (n = 3)
*CRLF1, EBI3,* *IL12B
Chemokine receptor activity (n = 24)
*ACKR2, ACKR3, ACKR4, CCR1, CCR10, CCR2, CCR3, CCR4, CCR5, CCR6, CCR7, CCR8, CCR9, CCRL2, CXCR1, CXCR2, CXCR3, CXCR4, CXCR5, CXCR6, GPR17, GPR35, GPR75, XCR1*
Chemokine and cytokine receptor activity (n = 2)
*CMKLR1, CX3CR1*

*GO:0008083, growth factor activity.

### Tissue-specific expression of cytokines

**Figure 2:**
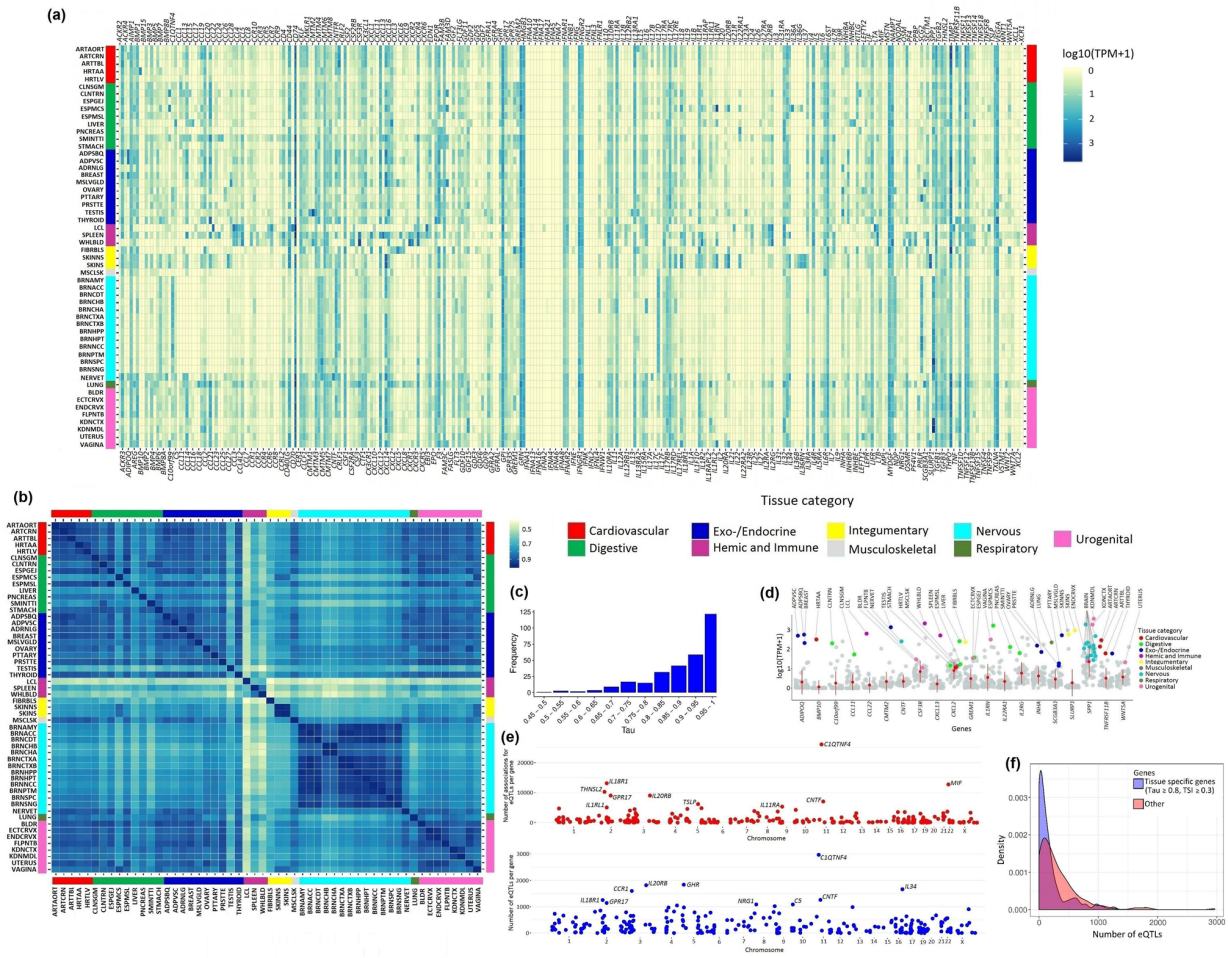
Expression profiles and genome-wide eQTL data for cytokines. The tissues are shown by abbreviations (Supplementary Table S2) and grouped by tissue categories (used throughout). (**a**) Gene expression profiles, built from TPM (Transcripts Per Million). (**b**) Spearman’s rank correlation matrix of tissue gene expression (heatmap). (**c**) Distribution of Tau (tissue specificity) scores. (**d**) Tissue-specific genes (Tau ≥ 0.8, TSI (Tissue Specificity Index) ≥ 0.3) with the highest levels of expression in corresponding tissues. Circles colored in accordance with the legend for tissue categories show TPM value for the tissue(s) with the highest level of expression for a given gene. Mean ± SD for TPM values across all tissues for a gene of interest is colored in red. Grey circles represent TPM values for a given gene in other tissues. Thirteen tissues from different brain regions are signed with a common label ‘BRAIN’. (**e**) Manhattan plot with the number of associations for eQTLs (Y-axis) targeting cytokines. Top ten genes are signed (upper panel). Manhattan plot with the number of eQTLs (Y-axis) for cytokines. Top ten genes are signed (bottom panel). (**f**) A density plot showing the distribution of the number of eQTLs per gene in the set of tissue-specific genes and in the set of ‘other’ genes.

In the GTEx V8 database^8^, we found 310 genes, among which four genes were not detected in any tissues and seven genes were expressed in a single tissue (Fig. 2a, Supplementary Table S2 for GTEx tissue abbreviations, Supplementary Table S3). Tissue-pairwise Spearman rank correlation of gene expression values showed positive correlation in expression (mean Spearman's rho = 0.79, range 0.40–0.99). The lowest levels of correlation were observed for hemic and immune-related cells and tissues (cells—EBV-transformed lymphocytes, spleen and whole blood) between themselves, and between other tissues (Fig. 2b). Using the expression specificity metric Tau^9^ and tissue specificity index TSI^10^, we classified 255 (82.3%) genes as tissue-specific (Tau ≥ 0.8) (Fig. 2c), of which 110 genes had TSI ≥ 0.3. Based on these thresholds, we determined 20 tissue-specific genes with the highest levels of expression in corresponding tissues (Fig. 2d).

### Genome-wide detection of cytokine eQTLs

Next, we performed the expression quantitative trait loci analysis, which revealed in total 322,020 associations for 84,904 eSNPs (SNPs reported as eQTLs) targeting cytokines (Fig. 2e, upper panel (associations), bottom panel (eSNPs)). Tissue sample volume correlated with the number of GTEx associations, number of unique eSNPs, number of target genes and number of target cytokine genes (Pearson correlation coefficient ranged from 0.88 to 0.92). Twenty seven genes appeared to be non-eGenes (their expression level was not associated with any SNP). The top five genes, which were regulated by the largest numbers of eSNPs included *C1QTNF4, GHR, IL20RB, IL34* and *CCR1* (Fig. 2e, bottom panel, Supplementary Table S1).

Interestingly, we found an inverse correlation between Tau and the number of eSNPs influencing the corresponding gene expression (Spearman's rho = − 0.390, two sided correlation *P* value = 1.43E−12). More in depth analysis of eQTL statistics revealed that the expression level of tissue-specific genes (Tau ≥ 0.8, TSI ≥ 0.3) was regulated by a lower number of eQTLs compared to ‘other’ genes (Mean ± SD: 178.81 ± 250.90 vs. 321.35 ± 376.84, Mann–Whitney U-test *P* = 1.49E−06, Kolmogorov–Smirnov (KS) test *P* = 2.80E−05). The top five genes regulated by the largest numbers of eSNPs appeared to be non tissue-specific. After the exclusion of these five genes from the sample ‘other genes’, the differences remained significant (Mann–Whitney U-test *P* = 5.51E−06, KS test *P* = 7.40E−05) (Fig. 2f). We noticed that among eQTLs available for 49 tissues, eQTLs targeting corresponding top tissue-specific genes were not identified in 31 tissues. In the set of 20 top tissue-specific genes, the largest numbers of eQTLs in corresponding tissues (tissue samples, n > 350) were found for the following genes: *SLURP1* (Skin—Sun Exposed*/*Skin—Not Sun Exposed, 684/291 eQTLs), *CNTF* (Nerve—Tibial, 669), *CMTM2* (Testis, 58), *ADIPOQ* (Adipose—Subcutaneous, 45), *TNFRSF11B* (Thyroid, 40) and *CCL11* (Colon—Sigmoid, 39 eQTLs). For other top tissue-specific genes, the number of eQTLs, if any, was small, e.g., *CXCL2* (Muscle—Skeletal, 14), *CSF3R* (Whole Blood, 13), *TNFRSF11B* (Artery—Tibial, 8). These SNPs did not represent single LD blocks, average r^2^ for eQTLs targeting these genes was, respectively, 0.38, 0.33 and 0.26.

### Direction of eQTL effects on target gene pairs

**Figure 3:**
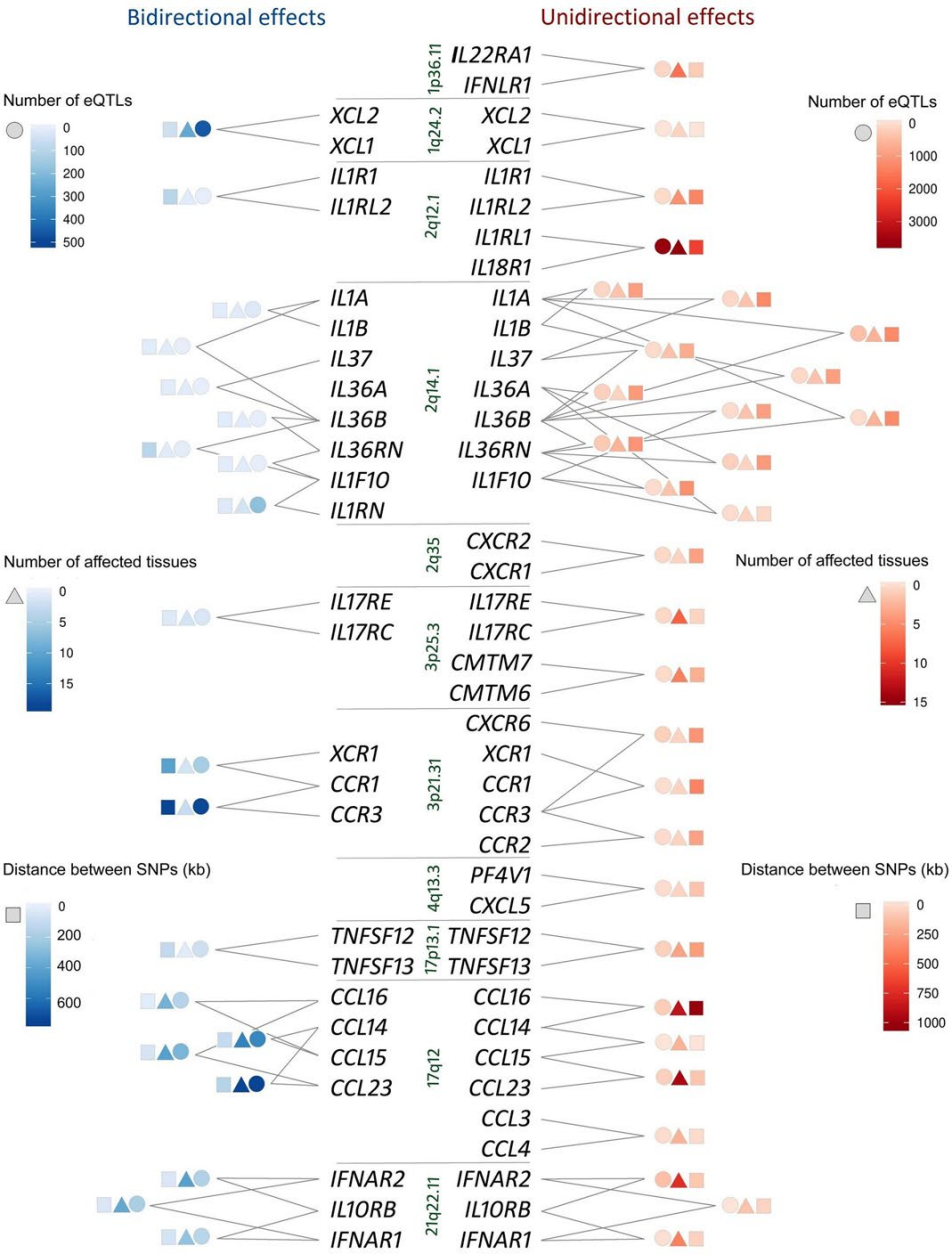
Direction of eQTL-gene pair effects. eQTL-gene pair effects are presented if the number of unidirectional or bidirectional associations for a given gene pair were ≥ 100. Genes are listed according their chromosome location. Bidirectional (left) or unidirectional (right) effects of eQTL-gene pairs are visualized by color. The effect information includes the number of eQTL-gene pairs (circles), the number of relevant tissues (squares) and the eQTLs distance (triangles).

A significant proportion of cytokines (n = 96) were located in gene clusters (Supplementary Table S4). Expression SNPs within these clusters were often associated with expression levels of more than one cytokine. We explored the direction of allelic effects of all eQTL-gene pairs within gene clusters. The majority of eQTL effects on a given cytokine pair in the same tissue were unidirectional (n = 95), however, opposite directional effects were also present (n = 51). For unidirectional and bidirectional eQTL effects, the numbers (mean ± SD) of associations and SNPs distances (kb) differed: 39.01 ± 231.81 versus 13.92 ± 60.83 (Mann–Whitney U-test *P* value = 2.15E−02) and 188.43 ± 212.04 versus 106.22 ± 133.67 (*P* = 2.87E−05). As expected, SNPs average LD metric r^2^ negatively correlated with a genomic distance (mean Spearman's rho − 0.56, one-sided *P* = 1.23E−11). For a given gene pair, the higher was the number of unidirectional associations, the lower was the number of bidirectional associations (if any) and vice versa (mean Spearman's rho − 0.23, one-sided *P* = 6.78E−03). Both unidirectional and bidirectional eQTL effects were registered in a wide spectrum of tissues. Gene clusters including gene pairs with more than one hundred shared eQTL-tissue associations are presented in Fig. 3. The largest number of unidirectional associations was revealed for *IL1RL1* and *IL18R1* genes (n = 3711). The largest number of bidirectional eQTL effects was detected in the cluster of chemokine ligand genes in the 17q12 region. Since cytokines are redundant in their activity, i.e., similar functions can be stimulated by different cytokines, we could assume that bidirectional effects of functionally related cytokines might partially neutralize each other, thus reducing the eQTL-attributed phenotypic diversity. However, multiple uni-and bidirectional associations were observed within the majority of gene clusters (Fig. 3, Supplementary Table S4), therefore the causality interpretation of potential phenotypic association should be translated from gene-specific to a gene cluster-specific level.

### Phenotypic spectrum of cytokine gene associations in the NHGRI-EBI GWAS catalog

**Figure 4:**
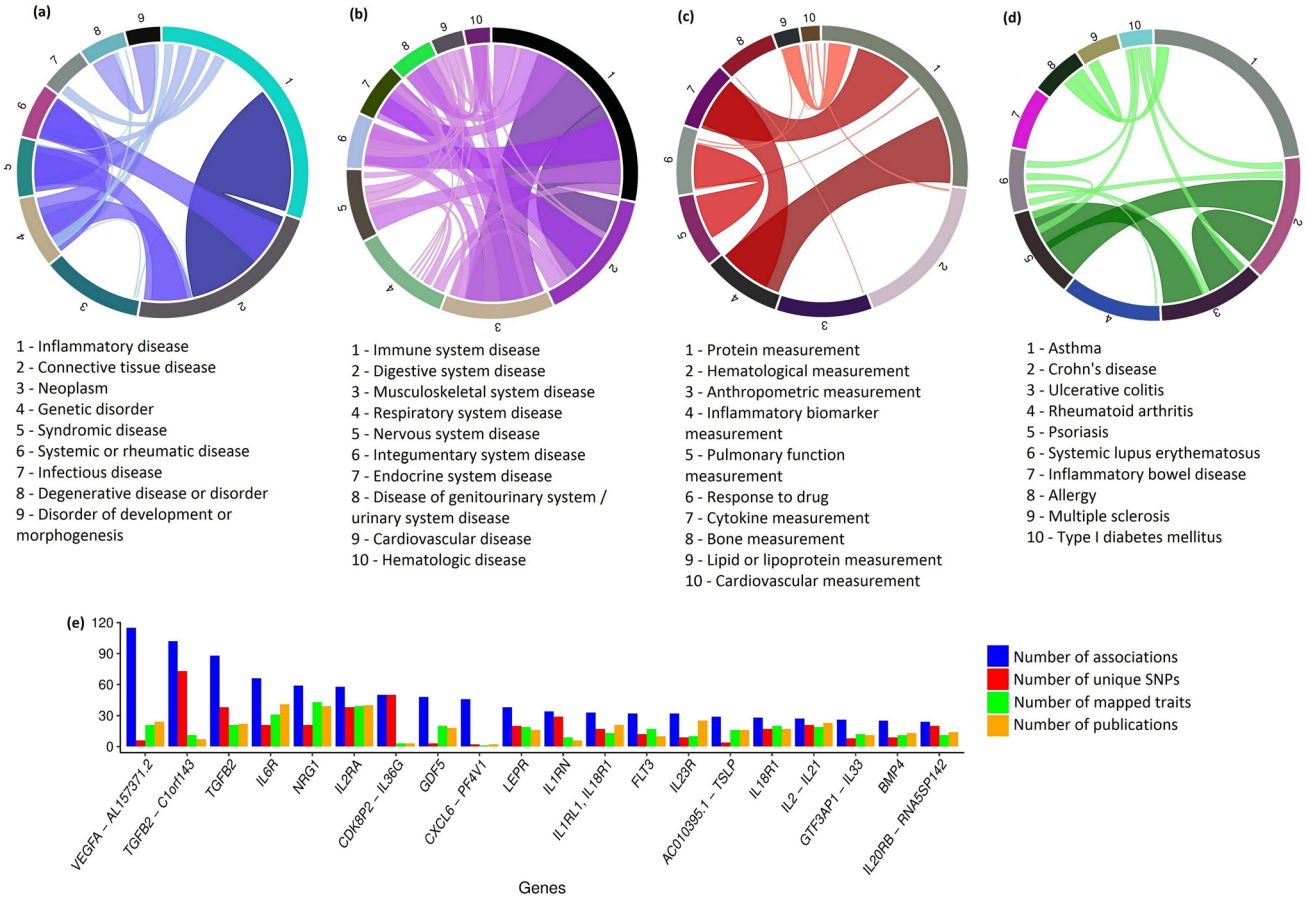
Representation of cytokine gene associations in the NHGRI-EBI GWAS Catalog. Circos plots show the proportions of top-ranked EFO (Experimental Factor Ontology) classifications for cytokines associations found in the NHGRI-EBI GWAS Catalog. These classifications were categorized by: (**a**) disease type, (**b**) disease by anatomical system (for non-oncological diseases), (**c**) type of measurement, (**d**) disease. The majority of the associations were described by several classification units, illustrated by the individual colored ribbons. Several associations were found in multi-trait studies (Supplementary Table S5). In figure panel (**d**), ribbons indicate associations that were both uniquely mapped and were studied together within the same framework. (**e**) Graph with top 20 genes by the number of corresponding associations. The numbers of unique SNPs, mapped traits and PubMed papers are also indicated.

In total, 244 cytokines were mapped in the NHGRI-EBI GWAS Catalog^11^ (Supplementary Table S5). Diseases and traits associated with cytokine genes were classified with the use of Experimental Factor Ontology (EFO)^12^. GWAS traits were annotated according to disease type, disease by anatomical system (for non-oncological diseases), and type of measurements (Supplementary Table S5, Fig. 4). The majority of the associations were described by several classification units. In the generated data sets of the most numerous categories (Fig. 4a–c) and diseases (Fig. 4d), the numbers of associations, SNPs and genes correlated (Pearson correlation coefficient range: set ‘disease type’ 0.93–0.99, set ‘disease by anatomical system’ 0.84–0.97, set ‘measurements’ 0.93–0.95, set ‘diseases’ 0.70–0.82). Based on the number of associations, we constructed one more set including 20 genes (Fig. 4e). In this set, the compared parameters strongly and disproportionately varied.

The highest numbers of associations covered by shared classifications were found for those related to inflammatory diseases and connective tissue diseases (Fig. 4a); immune and digestive system diseases, as well as immune and musculoskeletal system diseases (Fig. 4b); and protein measurements and inflammatory biomarker measurements (Fig. 4c). In the set of the top ten diseases, the comparison of disease pairs Crohn's disease—Ulcerative colitis and Crohn's disease (or Ulcerative colitis)—Psoriasis produced a similar number of shared associations (Fig. 4d). Infectious diseases were mainly represented by chronic infections. Only one study reported associations for acute infectious diseases^13^.

### The lists of GWAS trait-associated SNPs and their LD SNPs

**Figure 5:**
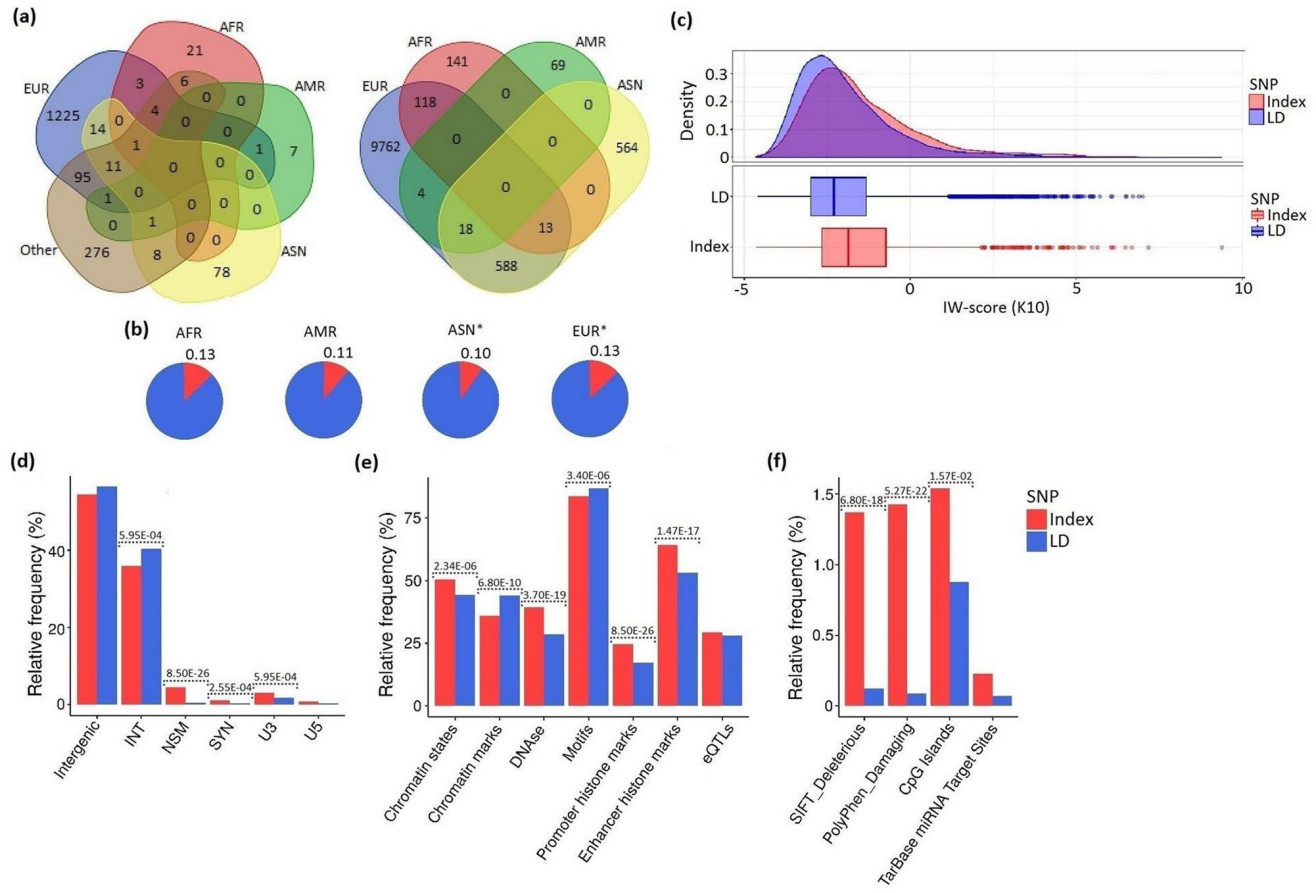
Population distribution and functional characterization of GWAS and LD SNPs. (**a**) GWAS-identified SNPs (left panel) and LD SNPs (right panel) by population are shown in a Venn diagram. In the population where GWAS (index) SNPs were detected, we selected SNPs in LD (linkage disequilibrium) with index SNPs using a threshold of r^2^ > 0.8. Only index SNPs were analyzed in mixed populations with different ethnicity or in populations, which could not be assigned to one of the four populations: EUR (European), ASN (East Asians), AFR (Africans including African Americans) and AMR (Admixed American). (**b**) Proportions of index and LD SNPs in the four populations. Asterisks indicate significant differences between EUR and ASN (*P* = 0.004) in the proportion of index and LD SNPs. (**c**) Density and box plots for functional IW-scores (K10) for index and LD SNPs. IW-scores (K10) were obtained from ten different scoring systems via IW-Scoring tool: an integrative weighted scoring framework to annotate and prioritize noncoding variations. (**d**) Distribution of genomic region annotations for index and LD SNPs (INT, intronic variant; NSM, non-synonymous missense variant; SYN, synonymous variant; U3, 3′untranslated region variant; U5, 5′untranslated region variant). (**e**) Distribution of potential regulatory sequences for index and LD SNPs. (**f**) Distribution of additional functional annotations for index and LD SNPs. SIFT and PolyPhen prediction scores were used to predict pathogenicity of amino acid substitutions. (**d**, **e**) Data were obtained from HaploReg v4; (**f**) Data were obtained from SNPnexus.

A total of 3077 associations for 1760 unique SNPs were found for cytokine genes in the NHGRI-EBI GWAS Catalog. Constructing a data set of cytokine SNPs, we included intragenic or intergenic SNPs in mapped (not reported) genes (Supplementary Table S5). We identified 891 intragenic SNPs and 869 intergenic SNPs, among the latter, 249 SNPs were located in regulatory regions. The majority of SNPs were obtained from Europeans. Next, we conducted an LD analysis using as criteria the threshold r^2^ > 0.8 (Fig. 5, Supplementary Tables S6, S7) and a clear identification of the population. The numbers and the proportions of index SNPs and LD SNPs in the studied populations are shown in Fig. 5a,b. Significant discrepancies in the proportions of index and LD SNPs were observed between EUR and ASN (*P* = 0.004). Other comparisons did not yield significant results due to smaller differences and/or smaller sample sizes.

### Functional annotation of index and LD SNPs

To compare functional characteristics of index and LD SNPs, we used IW-Scoring: an integrative weighted scoring framework to annotate and prioritize noncoding variations^14^ (Fig. 5c, Supplementary Tables S6, S7). Density and box plots demonstrated that index SNPs had much higher scores, i.e. higher functionality in comparison with LD SNPs (KS test *P* < 1.0E−06). A comparison of some other types of functional annotations via HaploReg v4 and SNPnexus (Fig. 5d–f, Supplementary Tables S6, S7) also mostly demonstrated a predominance of functional SNPs among index SNPs compared to LD SNPs. However, in individual pairs of index and LD SNPs, more functional LD SNPs were also observed. For example, top ten functional LD SNPs had an average IW-score 6.24, while their index SNPs had an average IW-score − 0.68.

### Natural selection analysis

**Figure 6:**
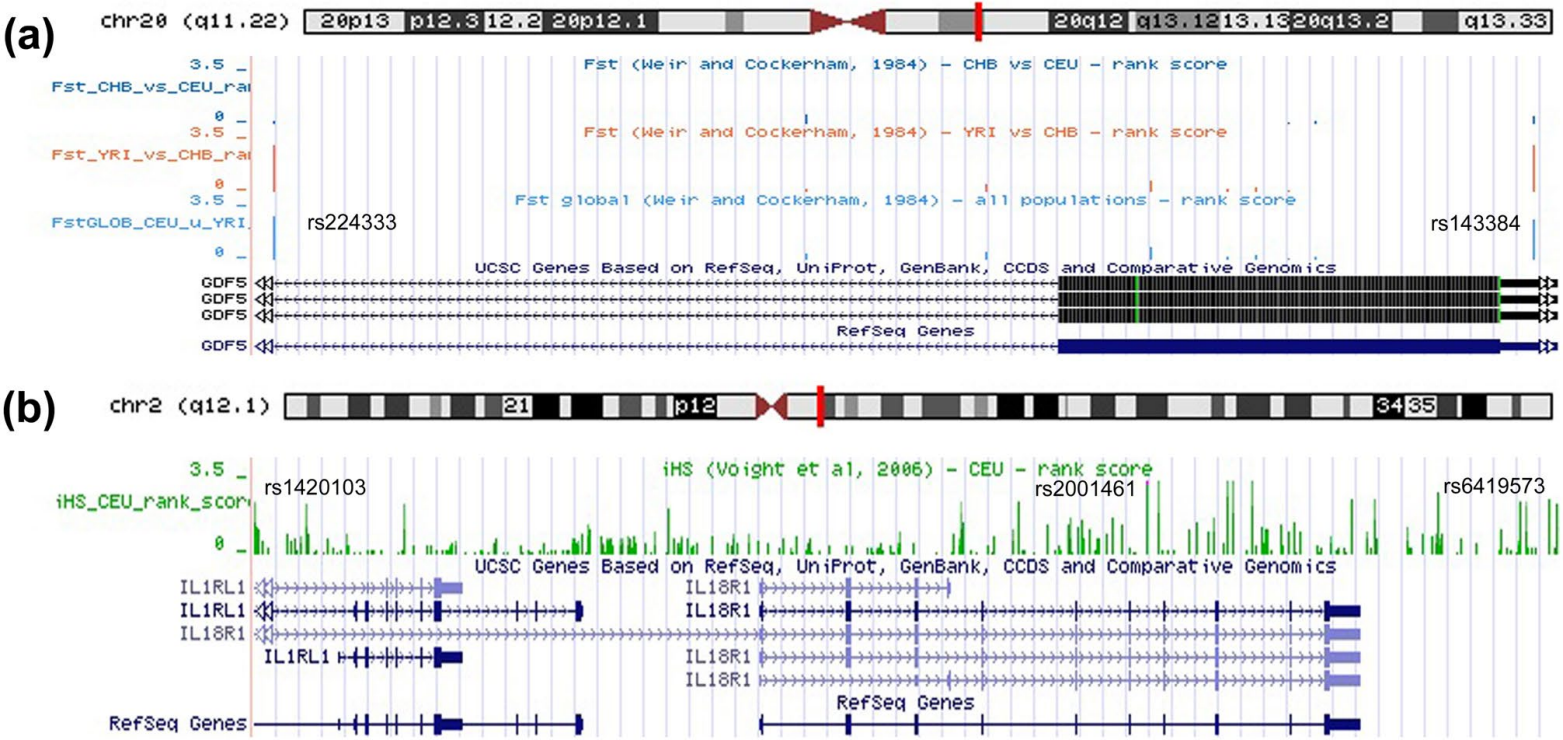
Evidence of natural selection for the GWAS SNPs in the *GDF5* and *IL1RL1/IL18R1* genes. Data were extracted from the 1000 Genomes Selection Browser 1.0 for two natural selection tests: global Fst and integrated haplotype score (iHS). (**a**) Global Fst values for the SNP rs143384 (Fst = 0.649) and rs224333 (Fst = 0.714) in the *GDF5* gene. GWAS signals for the SNPs rs143384 and rs224333 were reported for European and mixed ancestry populations. (**b**) iHS signals for the SNPs rs2001461 (iHS = 4.54), rs1420103 (iHS = 3.46) and rs6419573 (iHS = 3.34) in the *IL18R1*/*IL1RL1* locus in CEU population. GWAS signals for these SNPs were reported for European ancestry individuals.

Natural selection analysis was carried out for GWAS SNPs linked to cytokine genes. We used global Fst and integrated haplotype score (iHS) as measures of positive selection signals. The absolute scores and rank scores (− log10 of the *P* value centile rank of the SNP compared to others across the genome) were extracted from the 1000 Genome Browser^15^. Absolute scores having significant rank scores (> 2) are presented in Supplementary Table S8. It is accepted that SNPs with Fst scores ≥ 0.5^16^ or iHS scores ≥ 2.0^17^ are subjected to positive selection. All Fst and iHS signals with significant rank scores corresponded to these cutoff values. A total of 75 SNPs had global Fst rank scores > 2 (scores ranged from 0.404 to 0.668). Among them, the majority of GWAS associations, mostly with anthropometric measurements, were found for the two SNPs rs143384 and rs224333 in high LD (r^2^ = 0.93 in all populations) in the *GDF5* gene (Fig. 6a). Similar effects were also observed for *GDF5* rs143384 and rs224333 (scores ranged from 0.649 to 0.714) when comparing Fst CEU (Northern European) vs. YRI (West African). Five SNPs in high LD (r^2^ = 0.90 in all populations) in or near the *TGFB2* gene showed significant Fst values for the CEU-CHB (East Asian) pair (Supplementary Table S8). Only ten SNPs had rank scores > 2 for the iHS CEU score; among them three tightly linked SNPs (r^2^ = 0.86) were located in or near *IL18R1* gene (Fig. 6b). The top *IL18R1* SNP rs2001461 with an iHS CEU score of 4.544 was associated with blood protein (IL18R1) measurement, while two other SNPs were associated with serum ST2 (the *IL1RL1* gene product) measurement (rs1420103) and atopic eczema (rs6419573). Some other *IL1RL1/IL18R1* SNPs, which were not reported in GWAS Catalog studies also had high scores (Fig. 6b), however, this could be the result of hitchhiking effects^18^. The aforementioned SNPs were in low LD (r^2^ < 0.2) with top asthma-related SNPs widely represented in the region 2q12.1. Based on the results of the tests we used, the genes themselves were not shown to be under selection: *GDF5*, Global Fst 0.36; *TGFB2*, Fst CEU-CHB 0.06; *IL18R1*, iHS CEU 1.06.

Interestingly, SNPs under selection pressure were more often associated with different types of measurements in comparison with other GWAS SNPs (85.09%, 348 from a total of 409 associations vs. 70.27%, 1865 from a total of 2654 associations, *P* = 6.9E−10). Among the measurements, the most pronounced differences were related to anthropometric measurements (*P* = 1.4E−10). These differences were mainly due to the SNPs in or near the *TGFB2* gene (35 associations) and in the *GDF5* gene (35 associations). From the total of 77 associations with anthropometric measurements, 73 items were linked to height- and body mass index (BMI)-related phenotypes. Among five *TGFB2* SNPs, only rs1108548 had eQTL effects (two associations in the GTEx database). SNPs in the *GDF5* gene rs143384 and rs224333 had, respectively, 116 and 130 records in the GTEx database. The number of the GTEx associations for the *IL1RL1*/*IL18R1* SNPs (rs2001461, rs1420103, rs6419573) ranged from 35 to 41 items.

### eQTL analysis of GWAS Catalog SNPs in cytokine genes

**Figure 7:**
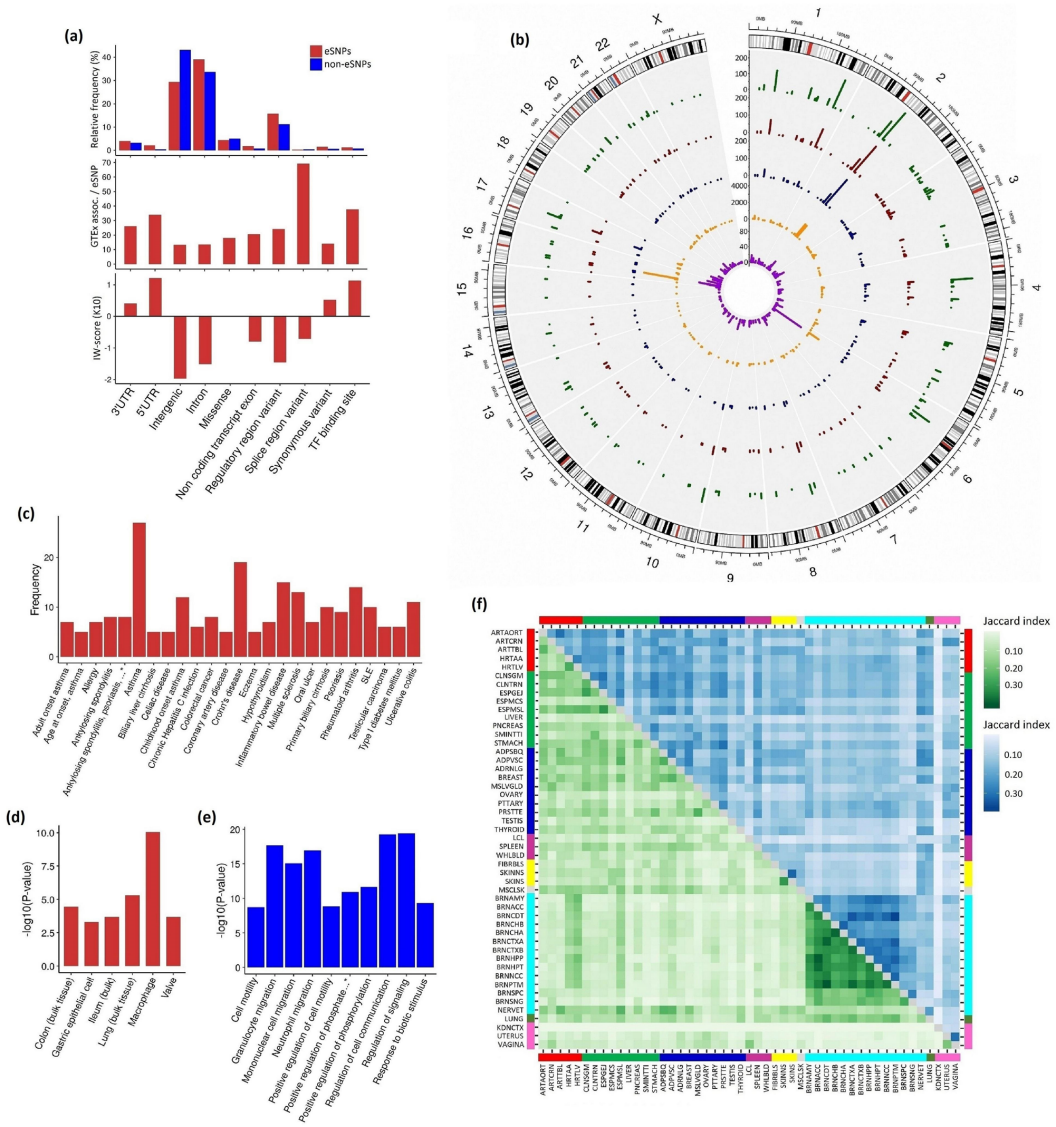
eQTL analysis of GWAS Catalog SNPs in cytokine genes. (**a**) Distribution of eSNPs and non-eSNPs by genomic region (upper panel). Number of GTEx associations per eSNP by genomic region (middle panel). IW-scores (K10) by genomic region (bottom panel). (**b**) Circos plot illustrating by chromosomal region distribution of the numbers of GWAS Catalog associations, unique GWAS Catalog SNPs, unique eSNPs, eSNP associations in the GTEx v.8 database and target genes (from the periphery to the center). (**c**) Spectrum of GWAS diseases associated with eSNPs in cytokine genes (frequency of association occurrence ≥ 5). (**d**) Results of ARCHS4 Tissues enrichment analysis for 655 target genes encoding proteins involved in protein interactions. (**e**) Top ten unique GO terms returned by GO enrichment analysis for the whole set of target genes. (**f**) Jaccard tissue similarity matrix for eQTL profiles based on matching eQTLs with their target genes and NES (Normalized effect size) direction: for the whole set of target genes (above the diagonal) and for the subset of target cytokines (under the diagonal). *(**c**) Ankylosing spondylitis, psoriasis, ulcerative colitis, Crohn's disease, sclerosing cholangitis; (**e**) positive regulation of phosphate metabolic process.

Our search of cis-eQTL SNPs yielded 19,386 associations for 1142 SNPs, targeting 999 genes, of which 178 genes represented cytokines (Supplementary Table S9). Compared to SNPs without eQTL effects (non-eSNPs), eSNPs were more often located in intergenic regulatory regions and in introns, while non-eSNPs were more frequently found in non-regulatory intergenic regions (Fig. 7a, upper panel). The most associations in the GTEx database, per one eSNP, were found for splice region variants, TF (transcription factor) binding site variants and 5′UTR variants (Fig. 7a, middle panel). In the set of eSNPs, positive IW-score (K10) mean values were revealed for TF binding site variants, synonymous variants, 5′UTR and 3′UTR variants (Fig. 7a, bottom panel). In the context of the direction of eQTL effects on target gene pairs, quite a lot of these effects were unidirectional for one gene pair and bidirectional for another gene pair. Both uni- and bidirectional associations were found for 78 eQTLs from, respectively, 188 and 103 eQTLs with unidirectional and bidirectional effects on target gene pairs.

The Circos plot demonstrating the numbers of GWAS Catalog associations, unique GWAS Catalog SNPs, unique eSNPs, eSNP associations in the GTEx v.8 database and target genes, depicted according to chromosome regions is provided in Fig. 7b. The largest number of eSNPs was reported for the region 2q14.1. For this region, we found 220 GWAS Catalog associations and 188 eSNPs targeting 27 genes, among them nine cytokines (*IL36RN, IL1A, IL37, IL1B, IL1F10, IL36A, IL36G, IL36B, IL1RN*). The majority (202/213) of eSNP associations were detected for different types of measurements, primarily, for interleukin-1 beta measurement. The largest numbers of eSNP disease associations were linked to the following regions: 2q12.1 (58 associations, 32 eSNPs), 10p15.1 (31 associations, 12 eSNPs), 1q21.3 and 5q22.1 (27 associations, 11 eSNPs and 5 eSNPs, respectively). The top five diseases associated with all GWAS Catalog eSNPs were: asthma, Crohn's disease, inflammatory bowel disease, multiple sclerosis and rheumatoid arthritis (Fig. 7c).

Next, we looked at the specificity of cytokine genes eQTLs for each trait in the GTEx panel (Supplementary Table S10). The most significant associations (*P*exp < E−10) were found for the following disease-tissue pairs: (1) cardiovascular, *IL6R* (coronary artery disease, atrial fibrillation, abdominal aortic aneurysm) and *BMP1 (*coronary artery disease); (2) digestive, *CCR1* (celiac disease), *TSLP* (eosinophilic esophagitis) and *IFNGR2* (ulcerative colitis, Crohn's disease, sclerosing cholangitis, inflammatory bowel disease); (3) endocrine, *NRG1* (hypothyroidism), *GFRA2* (type II diabetes mellitus) and *IFNGR2* (sclerosing cholangitis); (4) immune/hematologic, *CCL20* and *CXCL5* (inflammatory bowel disease, ulcerative colitis), *CCR1* (celiac disease, Behcet's syndrome, AIDS), *GDF15* and *IL12RB2* (systemic lupus erythematosus), *IFNGR2* (multiple sclerosis) and *TNFSF15* (Crohn's disease); (5) integumentary, *IFNLR1* (psoriasis) and *IL1A* (eczema); (6) musculoskeletal, *IFNGR2* and *IFNLR1* (ankylosing spondylitis); (7) nervous, *CCL20* (eating disorder), *FLT3* (Tourette syndrome), *IFNAR1* (narcolepsy with cataplexy), *IL18R1* (anterior uveitis and leprosy); (8) respiratory, *IL1RL1* and *IL18R1* (asthma). The subsequent analysis of GWAS disease-relevant tissue-specific eQTLs showed that eQTLs linked to nervous system diseases were enriched (had lower expression *P* values) in nervous compared to integumentary tissues; and eQTLs observed in cardiovascular diseases were enriched in cardiovascular against nervous tissues (Supplementary Table S10).

We further used the STRING database^19^ for two types of enrichment analysis. First, we retrieved protein–protein interaction information for a total of 999 target genes, of which 655 genes encoded proteins found to be involved in protein interactions (Supplementary Table S11). The list of 655 genes was analyzed for the enriched categories from the ARCHS4 tissue database via the Enrichr tool^20^. ‘Macrophage’ was the top term followed by five more tissues, namely, lung (bulk tissue), colon (bulk tissue), ileum (bulk), valve and gastric epithelial cell (Fig. 7d). Since interacting proteins participate in many functions determining, in particular, tissue phenotypes in health and disease^21^, the aforementioned results are in agreement with the fact that many GWAS Catalog diseases found for cytokine genes were represented by inflammatory diseases and by respiratory and digestive system diseases. Second, we performed GO enrichment analysis for the following gene sets: target cytokines (n = 178), target non-cytokine genes (n = 821) and the combined group of 999 genes. No enriched terms were retrieved for the group of non-cytokine genes. Enriched biological process (BP) annotations included 838 and 457 annotations for the set of cytokines and for the whole set, respectively (Supplementary Table S12). Among annotations found for the whole set, 57 annotations were unique, of which the top ten annotations were related to different types of regulation, including regulation of cell communication and migration (Fig. 7e). Subsequent processing of GO annotations, with the use of the REVIGO tool^22^, allowed selection of common and unique top-level functional categories for the set of cytokines and for the whole set (Supplementary Table S13). In the latter set, non-cytokine genes might contribute to cytokine network, in particular, participating in ‘apoptotic cell clearance’, since clearance defects are associated with the processes underlying inflammation and autoimmunity^23^.

Next, we used the Jaccard index to measure pairwise tissue similarity for eQTL profiles by matching eQTLs with their target genes and NES (Normalized effect size) direction. The Jaccard index was calculated for three sets: only cytokines as target genes for GWAS Catalog eSNPs, all target genes for our set of GWAS Catalog eSNPs, and cytokines as target genes at the genome-wide level (in total, targets for 84,904 eSNPs) (Supplementary Table S14). In the set of cytokines as target genes for GWAS Catalog eSNPs, the Jaccard index ranged from 0 to 42.33 (mean ± SD, 8.98 ± 8.00). It was somewhat higher when considering the whole pool of target genes (range 0.45 to 44.94, mean ± SD, 14.34 ± 6.78) (Fig. 7f). In the third set, the Jaccard index had values closer to those found for the first set (range 0.31–34.29, mean ± SD, 9.78 ± 6.15). Overall, the mean Jaccard index was low in all sets. The highest sharing of eQTLs across tissues was observed within the same tissue categories.

## Discussion

Gene lists of human cytokines vary from 132 to 261 genes depending on whether cytokine receptors are included^24^. Using the QuickGo database and the search terms ‘cytokine/chemokine activity’ and ‘cytokine/chemokine receptor activity’, we generated a list of 314 cytokines, which comprised an extensive range of cytokines/chemokines and their receptors. We aimed at collection, analysis and interpretation of data on cytokines focused on their tissue-specific expression, eQTLs and GWAS diseases and traits.

Our results on high tissue-specificity of cytokines are in agreement with the literature data. Inflammation initiation and resolution are mediated by pathways involving different cytokines, which work together with other tissue-specific signals depending on the composition of the relevant tissue and the microbial load^24,25^. The results of inverse correlation between cytokine tissue-specificity and the number of eQTLs targeting their expression should be assessed with caution since there is a positive correlation between the number of eQTLs and the tissue sample size, as well as gene length and the number of SNPs in tight LD with the top eSNP. Nevertheless, gene expression is a trait that is often under stabilizing selection, which plays an important role in limiting discrepancy in gene-expression levels^26,27^ substantial for the maintenance of tissue specificity in the expression regulatory framework^28^. Moreover, eQTLs can be predominantly targets of negative selection, in particular those affecting genes essential for tissue function, i.e. tissue-specific genes^29,30^. In this context, our results on a smaller number of eQTLs in tissue-specific cytokine genes, in comparison with other genes, seem biologically plausible. We also demonstrated many uni- and bidirectional changes in expression levels of target cytokine pairs associated with the same SNPs. A dominance of unidirectional correlations was seen in large gene clusters, mini-clusters and individual gene pairs. Gene clusters are regions of co-localized genes, which were formed in the course of evolution due to duplication of a single gene. The two newly formed copies usually developed specialized functions without losing a common primary function^31^. Cytokines gene clustering was intended to provide a consistent response to inflammatory stimuli^32^, while complex interplay of eQTLs influencing expression of genes in both directions could enable fine-tuning of the inflammatory response.

We classified all GWAS diseases and traits linked to cytokine SNPs with the EFO classification, described the top classification units for mapped traits and demonstrated their pairwise overlappings. The disease spectrum mainly included chronic immune-related disorders with a wide representation of autoimmune diseases, while associations found for infectious diseases, especially acute conditions, were relatively scarce.

It is accepted that GWAS-identified SNPs are usually considered as markers, and other SNPs in high LD with the index SNPs may be causal for the disease^33^. The comparative analysis of index SNPs and LD SNPs revealed higher functional scores for index SNPs, however, the influence of individual non-GWAS functional SNPs in different degrees of LD with the index SNPs can be essential. We reported a larger proportion of LD SNPs in Asians vs. Europeans. This finding may be explained by the fact that GWAS SNPs were more often located in intragenic regions in Asian (52.21%) than in European populations (46.27%), since an excess of SNPs in strong LD is an inherent property of intragenic SNPs^34^.

Immune-related genes and cytokines, in particular, are frequently targets for natural selection in humans^35^, therefore, we performed two natural selection tests, Fst and iHS, and found several SNPs subjected to high selective pressures. Given the results of the Fst test, the SNPs in or near the *GDF5* and *TGFB2* genes were mainly associated with anthropometric measurements related to height and BMI. Height is one of the best known candidates for polygenic selection in humans, especially in Europeans, while data for BMI are contradictory^36,37^. The *GDF5* gene product regulates bone and cartilage formation; recent selection of growth phenotypes affected *GDF5* alleles, which were also associated with an increased arthritis susceptibility, especially in East Asians^38,39^. In our study, the *GDF5* SNPs, rs143384 and rs224333, were associated with height- and BMI- related phenotypes in different populations, however, no associations with arthritis were revealed for these SNPs under selective pressure. The *TGFB2* gene product also has growth factor activity. According to GO annotations, it participates in skeletal system development. In our study, significant Fst results were observed only for the CEU-CHB pair of populations and the SNPs under selective pressure had almost no effect on the *TGFB2* expression. Thus far, we did not find literature data on the involvement of *TGFB2* SNPs in the selective processes. The iHS test, aimed at defining evidence of recent positive selection, detected selective sweeps for three tightly linked SNPs in the *IL18R1* gene, which encodes a cytokine receptor from the interleukin 1 receptor family. It has been previously discussed that the conserved across evolutionary branches mechanism of regulating IL18 signaling might represent a target for selective pressure^40^. This assumption is consistent with the fact that the top *IL18R1* SNP rs2001461 (iHS CEU score 4.54) was associated with the IL18R1 expression (GWAS trait *P* value = 3.00E−129).

Our eQTL analysis of GWAS SNPs revealed the largest number of associations in the GTEx database for splice region variants, TF binding site variants and 5′UTR variants, i.e., the regions functionally relevant to gene expression and its regulation^41^. The analysis of tissue specificity of cytokine genes eQTLs for each trait in the GTEx panel was aimed at highlighting the most significant findings for disease-tissue associations. Some eQTLs influenced the expression levels of many (more than 20) different cis-genes in multiple tissues thus complicating the eQTL data interpretation. The relevance of eQTLs may be supported by the results of the tissue- and disease-specific enrichment analysis^42^, however, the specific level of enrichment was observed in only two sets of comparisons. Lack of enrichment results for the majority of tissue-specific effects of eQTLs can be explained by a lack of statistical power and pleotropic effects of many SNPs. The true absence of tissue-specific effects for some complex traits is also discussed^43^. The role of eGenes represented by cytokines in comparison with the role of other eGenes was highlighted in the gene set enrichment analyses, which showed that cytokines were involved in infection and inflammation-related biological processes, while other genes in the whole set of target genes were mainly engaged in regulation, cell communication and migration. All together these data imply that cytokines expression variability fundamentally contribute to the molecular origins of complex traits and immune-mediated diseases. Tissue similarity in eQTL profiles of GWAS trait-associated SNPs measured by Jaccard coefficients showed high eQTL specificity. These results are in agreement with the fact that tissue-specific eGenes are more often annotated as disease genes than tissue-shared eGenes^42^. Tissue-specific genes are relevant to tissue biology and disease^29^. Among other regulatory mechanisms, cytokine eQTLs specificity could contribute to cytokine tissue expression specificity. The GTEx database provides data on tissue expression in healthy tissues, however, the validity of the approach using GTEx data for translational research in medical science has been recently confirmed in the study of drug targets, in which druggable genes were expressed in disease-relevant tissues in a healthy state in 87% of cases^44^.

The main limitations of this study are characteristic for secondary investigations using data as they are in original resources. The enrichment analysis of GTEx data was limited to the results presented with q-value threshold 0.05. GTEx eQTL effects may be gender- and age- dependent and linked to population structure, thus being subjected to confounding^7,45^.

In conclusion, we generated a list of 314 cytokines and characterized their tissue-expression specificity, eQTLs and GWAS diseases and traits. Several findings go beyond the scope of this study and may be interesting for future research directions. (1) Correlation between cytokine gene expression levels in different GTEx tissues was high, however, the lowest levels of correlations were revealed for whole blood and other hemic and immune-related cells and tissues, between themselves and between other tissues. These data are in agreement with GTEx data for the whole set of GTEx genes^7^ and suggest that using blood as a surrogate tissue for transcription analysis has marked limitations for translational research. (2) Low Jaccard index for eQTL-based tissue similarity reflects eQTL tissue-specificity. This conclusion is supported by literature data demonstrating that, if possible, disease-relevant tissue should be used for eQTL-based transcription analysis^46^. Other findings and observations are more specific to the aim of the study. (3) Acute immune-mediated conditions are scarcely represented among GWAS traits, possibly due to insufficient research and/or complexity and multifactoriality. (4) Natural selection analysis identified SNPs in the *GDF5* gene (confirmatory information) and *IL18R1* gene (new data) subjected to positive selection. (5) GWAS SNPs with eQTL effects affected expression levels of many eGenes in different tissues, however, it was cytokines expression variability that fundamentally contributed to the molecular origins of considered immune-mediated conditions.

## Materials and methods

### Hand-curated list of genes encoding proteins with cytokine and cytokine receptor activity

We generated a list of genes encoding proteins with cytokine/chemokine and cytokine/chemokine receptor activity employing the QuickGo database—a web-based tool of the European Bioinformatics Institute (EMBL-EBI) for Gene Ontology searching^7^. Our searching for the terms cytokine activity (GO:0005125), chemokine activity (GO:0008009), cytokine receptor activity (GO:0004896) and chemokine receptor activity (GO:0004950) provided 40,607, 11,241, 29,071 and 9474 annotations respectively (last access August, 2019). After the removal of non-human taxon entries, proteins without annotations in SwissProt and entries without a gene symbol approved by the HUGO Gene Nomenclature Committee, we constructed a final set of 314 unique genes.

### GTEx information on cytokine genes in the whole genome context: data extraction and analysis

For our gene-set analyses, we downloaded results from the GTEx database Analysis Release V8^8^. Gene tissue expression data presented as median TPM (Transcript Per Million) were available for 54 tissues (in total 948 donors). Cis-eQTL information was provided for 49 tissues (in total 838 donors) with significant eQTL signals determined with a Q-value threshold. Two metrics estimating tissue specificity were applied, Tau^9^ and TSI^10^, both calculating tissue specificity based on the information of a given gene expression in each tissue and its maximal expression across all tissues. Both metrics vary from 0, indicating that expression is constant in all tissues, to 1, pointing out that expression is specific to a single tissue.

To assess the direction of an eQTL effect on target gene pairs, we formed clusters of physically co-localized genes for a given chromosome region. These clusters included gene pairs and SNPs matched by tissue-specific expression. LD analysis was carried out by calculating the squared correlation coefficient (r^2^) for each pair of SNPs of interest. LD patterns were analyzed in populations of European descent (CEU, UtAh residents from North and West Europe; TSI, Toscani in Italia; FIN, Finnish in Finland; GBR, British in England and Scotland; IBS, Iberian population in Spain). LD analyses in this section and throughout the study were done with the LDlink resource^47^.

### The NHGRI-EBI GWAS Catalog data extraction and analysis

We extracted data for cytokine genes from the NHGRI-EBI GWAS Catalog (last access August, 2019)^11^. Associations reported in the GWAS Catalog were annotated with the use of EFO (Experimental Factor Ontology)^12^. We used HaploReg v4^48^ to construct two data sets including information for GWAS SNPs (index SNPs) and SNPs in high LD with the index SNPs. This information was obtained via haploR package^49^. LD SNPs were selected based on a threshold r^2^ > 0.8 and were matched by population with index SNPs. Only index SNPs were considered for mixed and non-indicated populations, as well as for populations which could not be attributed to any of the HaploReg populations: European, Asian (Chinese, Japanese, Vietnamese), African (including African Americans) and American (Admixed American). To annotate index and LD SNPs we used the IW-scoring tool, which was developed to annotate and rank non-coding genetic variants by their putative functional importance^14^. Among available outputs, we focused on IW-score (K10), which aggregates scores from ten state-of-the-art functional prediction tools for known genetic variants. We also considered some additional annotations provided by HaploReg v4^48^ and SNPnexus^50^ for non-coding and coding variants (annotations at the genomic region level, as potential regulatory sequences and the effect of the amino acid change on protein function).

### Detection of signals of positive selection

Two measures of positive selection signals for GWAS SNPs in cytokine genes, Fst (Fixation index) and iHS (Integrated Haplotype Score) were subjected to analysis via the 1000 Genome Selection Browser 1.0^15^. This resource includes data on populations of West African (YRI), Northern European (CEU) and East Asian (CHB) ancestry. Natural selection statistics are provided as the absolute scores and rank scores representing − log10 of the *P* value at 0.01 FDR for the SNP compared to others in the whole-genome context. Rank scores > 2.0 are considered significant^15^. Six sets, Fst CEU versus CHB, Fst CEU versus YRI, Fst Glob, iHS CEU, iHS CHB and iHS YRI were considered.

### Functional analysis of GWAS SNPs with eQTL effect

To investigate GWAS SNPs affecting gene expression levels, we also used the GTEx database Analysis Release V8. Among target genes, we identified gene sets significantly enriched in protein–protein interactions (PPI) and GO (Gene Ontology) terms in the STRING database^19^. For each PPI pair, the combined score > 0.4 was applied as the cutoff criterion. In the set of GO terms we included only terms with at least three genes per category. The resulting gene set was analyzed for tissue specific enrichment by ‘ARCHS4 Tissues’ in Enrichr^20^. For gene set enrichment analyses we set the false discovery rate (FDR) threshold as 0.05. The REVIGO tool was applied to remove redundancies in GO terms and to select the cluster GO representatives^22^. Tissue-sharedness in eQTL effects was assessed with the Jaccard similarity index, which measures similarity between two sets as the ratio of their intersection to their union. Tissue pairwise overlap was registered for eQTLs if they influenced the same target genes with the same direction of the effect.

### Statistical analysis

For categorical variables, we used Pearson's chi-square with Yates's correction/Fisher's exact test and displayed *P* values corrected for multiply testing (FDR test). For continuous variables, we applied two nonparametric tests, the Mann–Whitney U test and the KS tests. The Mann–Whitney test computes a *P* value depending on the discrepancy between the mean ranks of the compared groups, while the KS test compares the cumulative distribution of the two data sets. The Mann Whitney test is more appropriate for sample sizes < 50 samples.

### Ethics statement

Ethical review and approval was not required for the secondary analysis of public data in accordance with the local legislation and institutional requirements.

## Supplementary information

Supplementary Legends.

Supplementary Table 1.

Supplementary Table 2.

Supplementary Table 3.

Supplementary Table 4.

Supplementary Table 5.

Supplementary Table 6.

Supplementary Table 7.

Supplementary Table 8.

Supplementary Table 9.

Supplementary Table 10.

Supplementary Table 11.

Supplementary Table 12.

Supplementary Table 13.

Supplementary Table 14.

## Data Availability

All data generated or analyzed during this study are included in this article and its supplemental files.
