# Supplementary material for: Cytokines mapping for tissue-specific expression, eQTLs and GWAS traits
Source: Sci Rep. 2020 Sep 7;10:14740. doi: 10.1038/s41598-020-71018-6 (PMC7477549; doi:10.1038/s41598-020-71018-6)
Supplement: Supplementary file 1 — Supplementary Legends. [file 41598_2020_71018_MOESM1_ESM.docx]

**Cytokines mapping for tissue-specific expression, eQTLs and GWAS traits**

Lyubov E. Salnikova, PhD, Dr. Sci. (Biol.)^a,b*^, Maryam B. Khadzhieva, PhD^a,b^, Dmitry S. Kolobkov^a,c,d^, Alesya S . Gracheva, PhD^a,b^, Artem N. Kuzovlev, PhD, MD^b^, Serikbay K. Abilev, PhD, Dr. Sci. (Biol.)^a^

^a^Laboratory of Ecological Genetics, N.I. Vavilov Institute of General Genetics, Russian Academy of Sciences, 3 Gubkin Street, Moscow 117971, Russia

^b^Federal Research and Clinical Center of Intensive Care Medicine and Rehabilitology, Petrovka str, 25, b.2, Moscow 107031, Russia

^c^Department of Computer Science and Applied Mathematics, Weizmann Institute of Science, 234 Herzl St., PO Box 26, Rehovot 7610001, Israel

^d^Department of Molecular Cell Biology, Weizmann Institute of Science, 234 Herzl St., PO Box 26, Rehovot 7610001, Israel

Correspondence to: Lyubov E. Salnikova, Institute of General Genetics, Russian Academy of Sciences, 3 Gubkin Street, Moscow 117971, Russia, phone 74991328958, fax 74991328962

E-mail: [salnikovalyubov@gmail.com](mailto:salnikovalyubov@gmail.com)

**Supplementary Table Legends**

**Table S1.** The list of cytokine and cytokine receptor genes with information on their genomic location, number and by tissue distribution of eQTLs associated with the expression of these genes.

**Table S2.** GTEx tissue abbreviations.

**Table S3.** Median gene-level TPM (Transcripts Per Million) by tissue and tissue specificity indices Tau and TSI. The tissues are grouped by tissue categories. Sample sizes for each tissue are provided.

**Table S4.** Direction of eQTL-gene pair effects. Columns indicate the chromosomal region, target cytokines (cytokine 1 and cytokine 2), their genomic coordinates, the total number of eSNPs, the number of associations in the GTEx database and information on unidirectional and bidirectional eQTL effects, namely: the number of intersecting eQTL-tissue associations, the number of tissues, tissues names, the SNPs number, their chromosomal location and length (bp) along with average r^2^.

**Table S5.** The NHGRI-EBI GWAS Catalog associations for cytokine genes. Associations are linked with EFO (Experimental Factor Ontology) classifications within three categories: disease type, disease by anatomical system (for non-oncological diseases) and type of measurements. Data on the number of SNP associations in the GTEx database (if any) are presented.

**Table S6.** By population distribution and functional characterization of GWAS (index) SNPs presented as merged functional annotations from HaploReg **v**4 (columns A-AF), SNPnexus (AG-AJ) and SNPnexus IW-Scoring (AK-AL) tools.

**Table S7.** By population distribution and functional characterization of LD SNPs presented as merged functional annotations from HaploReg **v**4 (columns A-AF), SNPnexus (AG-AJ) and SNPnexus IW-Scoring (AK-AL) tools. LD SNPs were selected based on a threshold r^2^ > 0.8 and were matched by population with index SNPs.

**Table S8.** Positive selection signals for GWAS SNPs in cytokine and cytokine receptor genes from the 1000 Genome Selection Browser 1.0 ([http://hsb.upf.edu](http://hsb.upf.edu/)). Absolute scores and rank scores for the SNPs with rank scores > 2.0 are presented for two different natural selection tests, Fst (Fixation index) and iHS (Integrated Haplotype Score).

Abbreviations: Han Chinese in Beijing, China (CHB), Yoruba in Ibadan, Nigeria (YRI), Utah Residents with Northern and Western European Ancestry (CEU)

**Table S9.** eQTL data for GWAS Catalog SNPs in cytokine genes.

**Table S10.** Tissue specificity of cytokine genes eQTLs for each trait in the GTEx panel. **(Table Sheet 1)** Diseases and tissues were grouped according to the classification by anatomical system and tissue categories. For each series of disease-tissue pairs, we presented data on eSNPs, their top target genes with expression *P* values and the lists of other target genes.  If the same eQTL influenced the expression of the gene in several tissues within the tissue category, the most significant category-specific *P* value was chosen for the analysis. From a group of SNPs in LD with r^2^ ≥ 0.8 (in Europeans), only the top SNP (by *P*exp in corresponding tissue) were included. For easy comparison, data in all tissue categories are shown. (**Table** **Sheet 2**) The results of the enrichment analysis of GWAS disease-relevant tissue-specific eQTLs. GTEx category-specific *P* values for the eSNPs associated with GWAS disease groups were compared with the use of the Wilcoxon rank-sum test and the Kolmogorov-Smirnov test. Inclusion criteria: the number of disease-relevant tissue-specific eQTLs ≥ 10. Results were considered significant (in bold) at 0.01 FDR in either of the two tests.

**Table S11.** The results of protein-protein interaction analysis of target genes from the STRING database (<https://string-db.org/>). Threshold: combined score ≥ 0.4.

**Table S12**. The results of the target gene-set enrichment analysis from the STRING database (<https://string-db.org/>). Two gene sets were analyzed: the whole set of target genes (n=999) and the subset of target cytokine genes (n=178). Thresholds: the number of genes per category ≥ 3, FDR (False Discover Rate) < 0.05.

**Table S13.** Cluster GO representatives for GO term sets summarized with the REViGO tool (<http://revigo.irb.hr/>). Threshold: dispensability ≤ 0.05.

**Table S14.** Tissue pairwise Jaccard index based on matching eQTLs with their target genes and NES (Normalized effect size) direction. Three sets were analyzed: (1) whole set of target genes, (2) subset of target cytokines and (3) cytokines as target genes at the genome-wide level.
